# Effect of various heat treatment methods and optimization of their parameters on mechanical properties of AISI 4140 steel

**DOI:** 10.1038/s41598-025-17299-1

**Published:** 2025-08-29

**Authors:** Sharan Mudda, Ananda Hegde, Sathyashankara Sharma, B. M. Gurumurthy, Manjunath Shettar, M. C. Gowrishankar

**Affiliations:** Department of Mechanical and Industrial Engineering, Manipal Institute of Technology, Manipal Academy of Higher Education, Manipal, 576104 Karnataka India

**Keywords:** AISI 4140 steel, Optimization, Regression analysis, Impact energy, Engineering, Materials science, Mathematics and computing

## Abstract

AISI 4140 steel is one of the important category in the steels with wide range of applications including but not limited to automotive, general machinery, oil and gas industry. In the current study, an effort is made to understand the effects of heat treatment parameters, such as heat treatment temperature and holding time, on the mechanical properties of AISI 4140 steel, and to optimize these parameters to obtain the superior combination of mechanical properties. The three important heat treatments which are used in this study are annealing, normalizing and oil quenching. The heat treatment parameters such as temperature and time are varied at three different levels of 900, 925, and 950 °C, and 1, 1.5, and 2 h respectively. Using the full factorial method, total 9 experiments were carried out with all the possible combination of temperature and time as the variants. In each of the tests, hardness and impact energy values were evaluated using appropriate tests, while microstructural changes were analyzed through a scanning electron microscope (SEM). The results obtained through statistical analysis have shown that combination of 900 °C with 2 h for annealing, 919 °C with 2 h for normalizing and 944 °C with 1 h for oil quenching as the optimum combination of heat treatment parameters for superior combination of hardness and impact energy. Results showed that increasing temperature led to grain coarsening, reducing hardness but improving impact energy. Regression equations generated in this study which have R square value more than 90% may be used to predict the hardness and impact energy for any value of temperature and time which is within the range of values considered for this study.

## Introduction

AISI 4140 is commonly used in the manufacturing of structural components that demand high strength, toughness, and wear resistance. This steel is widely used in automotive, aerospace, defense, and industrial machinery, where mechanical reliability under dynamic loading conditions is needed. Its mechanical properties can be significantly enhanced with appropriate heat treatment processes, which alter the microstructure and change the mechanical properties such as hardness and impact energy. AISI 4140 is an alloy steel containing 1.36% Cr and 0.215% Mo, known for its good strength and fatigue resistance. Heat treatment plays a critical role in controlling the final properties of steels. Processes such as annealing, normalizing and oil quenching are commonly used to achieve desired combinations of hardness and toughness. It may be noted that EN19 and 42CrMo4 steels, which are similar in mechanical performance but differ in chemical composition and standards, have been considered as equivalent steels to AISI 4140, only in application context and not in strict metallurgical terms, for the sake of literature review.

A lot of research has been carried out to study the effects of various heat treatment processes on mechanical properties of AISI 4140 steel. Kandpal et al.^1^ found that hardening produces a martensitic structure, increasing hardness, annealing produces a ferritic structure with higher impact energy but lower hardness, while normalizing results in a ferrite pearlite mix that has a balance in toughness and hardness.

To study the effects of quenching on AISI 4140 steel, Basori et al.^2^ quenched the steel at varying holding times, in the same media at a fixed cooling rate, and found that as the holding time increased there was a reduction in retained austenite and refinement in the martensite structure, that led to an increase in hardness from 215 VHN to 545 VHN. To study the effects of various heat treatments on AISI 4140 steel, Bhagyalaxmi et al.^3^ carried out annealing, hardening and tempering heat treatments and found that hardening gave highest hardness, tempering improved tensile strength, and annealing had the highest ductility. Mahmood et al.^4^ found that slower air-cooling rates enhance strength and tolerance, while faster cooling rates increase hardness. When Austempering heat treatment was carried out at higher temperatures for AISI 4140 and 4340 steels, by Bilal et al.^5^, at higher austempering temperatures, due to the formation of a coarse bainite martensite structure, there was an increase in hardness and impact energy for both the steels, while lower temperatures resulted in fine bainite martensite structures which offered better balance in strength and toughness. Similarly, when Badaruddin et al.^6^ carried out austempering heat treatment on AISI 4140 steel, it was found that austempering at 362 °C formed a bainite-martensite- retained austenite structure with high tensile strength and strong fatigue growth resistance, and multi austempering further improves toughness due to formation of thicker retained austenite and bainitic structure. It has also been found that double quenching and tempering improved the impact energy of the steel, without significantly affecting hardness or strength^7^. Laxmi et al.^8^ and Passanha et al.^9^, showed that increasing tempering time, temperature or modifying quenchant oil viscosity, improved impact energy but reduced hardness, with optimal results obtained at mid-range tempering values and moderate oil viscosity. It has generally been observed that quenching and tempering leads to development of tempered martensite, which improves the hardness, tensile strength and corrosion resistance of the steel^10^. However longer holding times, during heat treatment, tend to reduce the hardness and corrosion resistance of the steel due to carbide precipitation and resulting microstructural changes^11^. Chuaiphan et al.^12^, who experimented on usage of AISI 4140 as base material for cane harvester cutter, by enhancing its properties through heat treatment, found that water quenching followed by tempering resulted in a bainite martensite structure with improved hardness and a balaned impact toughness. Zhang et al.^13^ carried out quenching of AISI 4140 steel and found that one of the reasons quenching leads to low impact toughness is because of high hydrogen absorption after austenitization, which results in quench cracks. The effects of quenching in various media on the properties of AISI 4140 was studied by Bhagyalaxmi et al.^14^, and it was found that while water quenching gave the highest hardness, castor oil gave the best overall performance with a balance in hardness, tensile strength and ductility.

Badaruddin et al.^15^ carried out annealing and quenching heat treatments on AISI 4140 steel, and found that annealed samples exhibited lower strength but greater ductility and lower cycle fatigue (LCF) life compared to the quenched tempered samples, due to the ferrite pearlite structure that resists cyclic crack growth better than martensite, thus making annealing more suitable for when the steel has to be used for LCF critical applications. Sharma et al.^16^ found that when AISI 4140 steel was subjected to dual phase and austempering heat treatments, austempering, due to formation of ferrite and cementite structure, gave highest strength and impact energy, while dual phase heat treatment resulted in ferrite martensite structure, which improved strength but lowered ductility. Jami et al.^17^ reported a 35% increase in hardness after oil quenching and tempering (300–400 °C), with highest hardness and torsional strength observed at 300 °C. Saeidi and Ekrami^18^ found that a bainite–ferrite microstructure offered higher impact energy and ductility than martensite-based structures at similar hardness.

Other than conventional heat treatments, research has also been done on the effect of non-conventional heat treatments on mechanical properties of AISI 4140 steel. Senthilkumar et al.^19^ found that deep cryogenic treatment at −196 °C for 24 h improved wear resistance of the steel by 215% oer conventional heat treatment, due to austenite to martensite formation.

Based on the literature review, we concluded that a substantial amount of work has been carried out to study the effects of various heat treatments on mechanical properties of AISI 4140 structural steel, but very little work has been done with regards to optimization of the heat treatment parameters to get the best combination of hardness and impact energy for the steel. This study aims to perform a comparative analysis of the heat treatment behavior of AISI 4140 steel. The objective is to evaluate the influence of annealing, normalizing, and oil quenching on the microstructure, hardness, and impact energy of the steel. Additionally, optimization techniques are applied to determine the best set of heat treatment parameters that maximize mechanical performance. The findings are expected to support better material selection and processing decisions in industrial applications where a balance between strength and toughness is crucial.

## Materials and methodology

### Material procurement and specimen Preparation

**Fig. 1:**
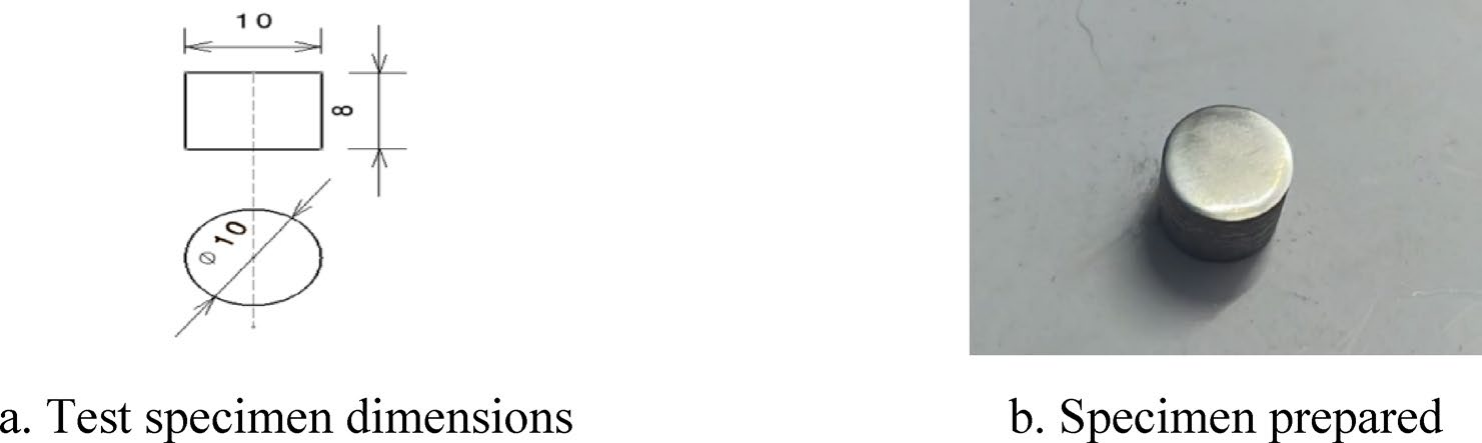
Microhardness and SEM analysis specimen as per ASTM E384 standards^20^.

**Fig. 2:**
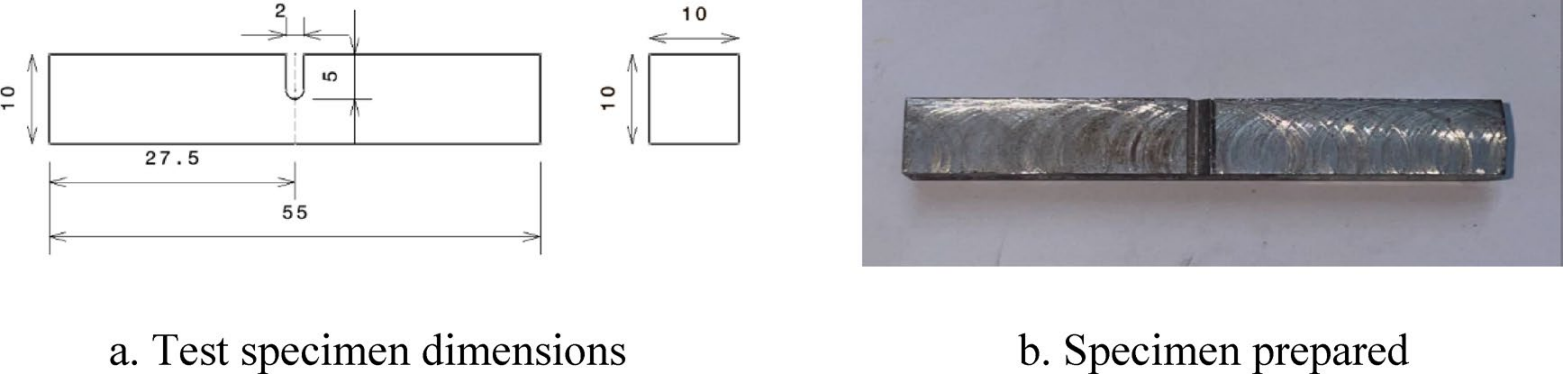
Charpy impact test specimen prepared as per ASTM E23 standards^21^.

AISI 4140 steel rods of 16 mm diameter were procured and machined to the required test specimen dimensions as per ASTM standards (as shown in Figs. 1 and 2) for both Vickers microhardness, SEM analysis and Charpy impact test. The specimens were prepared using lathe, CNC milling, wire EDM and slot milling machines.

### Spectroscopy analysis

Spectroscopy analysis, as shown in Table 1, was carried out to make sure that the material composition of the procured AISI 4140 steel was as per AISI standards.

**Table 1:** Spectroscopy analysis of AISI 4140 steel.

Element	C	Si	Mn	*P*	S	Cr	Ni	Mo	Fe
wt% obtained	0.417	0.243	0.66	< 0.001	0.012	1.36	0.096	0.215	96.8
wt% as per standards^22^	0.35–0.45	0.15–0.30	0.50–0.80	< 0.035	< 0.040	0.90–1.50	-	0.20–0.40	96.785–97.77

### Heat treatment

Separate specimens were prepared for annealing, normalizing and oil quenching heat treatments. The specimens are heated in a muffle furnace for austenitization heat treatment temperatures and soaking time conditions as mentioned in Table 2. The heat treatment temperature and holding time values as shown in Table 2 were chosen based on literature review^4^ and preliminary trials, wherein full austenitization and homogeneity was obtained.

**Table 2:** Heat treatment parameters.

Temperature (°C)	900			925			950		
Time (h)	1	1.5	2	1	1.5	2	1	1.5	2

**Fig. 3:**
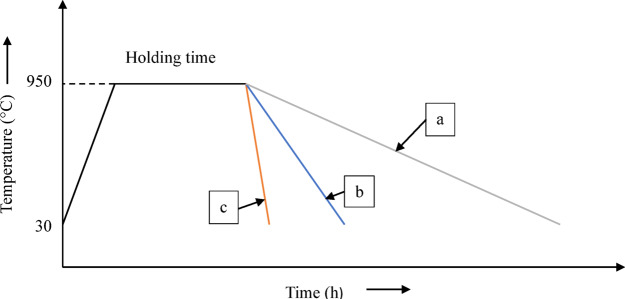
Heat treatment cycle (**a**) Annealing, (**b**) Normalizing and (**c**) Oil quenching.

Full factorial method was adopted to carry out the heat treatments by covering all the possible 9 combination of temperatures and time values. After heating, the annealing specimens were furnace cooled, normalizing specimens were air cooled, and oil quenching specimens were quenched in SAE 40 engine oil. SAE 40 is a single-grade oil with high viscosity at elevated temperatures, kinematic viscosity of 14 to 16 mm^2^/s at 100 °C, making it suitable for moderate quenching applications. It provides a slower cooling rate compared to water but faster than polymer-based or air cooling, helping to reduce distortion and cracking in medium-carbon steels. The cooling rate of three heat treatment processes is represented graphically in temperature vs. time graph in the Fig. 3.

### Mechanical characterization tests

ASTM E384-22^20^ was used as the standard for carrying out Vickers microhardness tests for assessing the hardness of all the test samples.

Charpy impact testing was carried out at the Advanced Composite Lab, Department of Aeronautical and Automobile Engineering, MIT, Manipal. Specimens were mounted in the testing machine, and a 300 J pendulum strike was applied. The energy absorbed during fracture was recorded to evaluate impact resistance. Testing followed ASTM E23^21^ guidelines.

For microstructural analysis, EVO MA18 SEM with Oxford EDS X-act, available at the Central Research Facility, MIT Manipal, was used.

## Result and discussion

### Mechanical characterization results

**Fig. 4:**
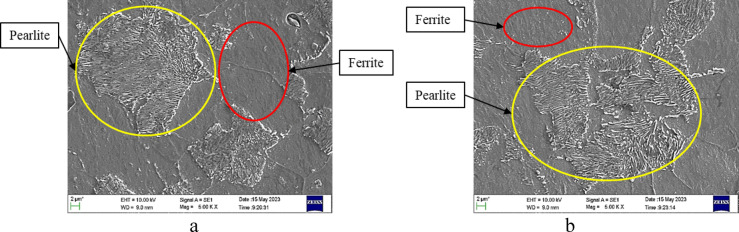
SEM image of AISI 4140 steel annealed at (**a**) 900 °C for 2 h and (**b**) 950 °C for 2 h.

**Fig. 5:**
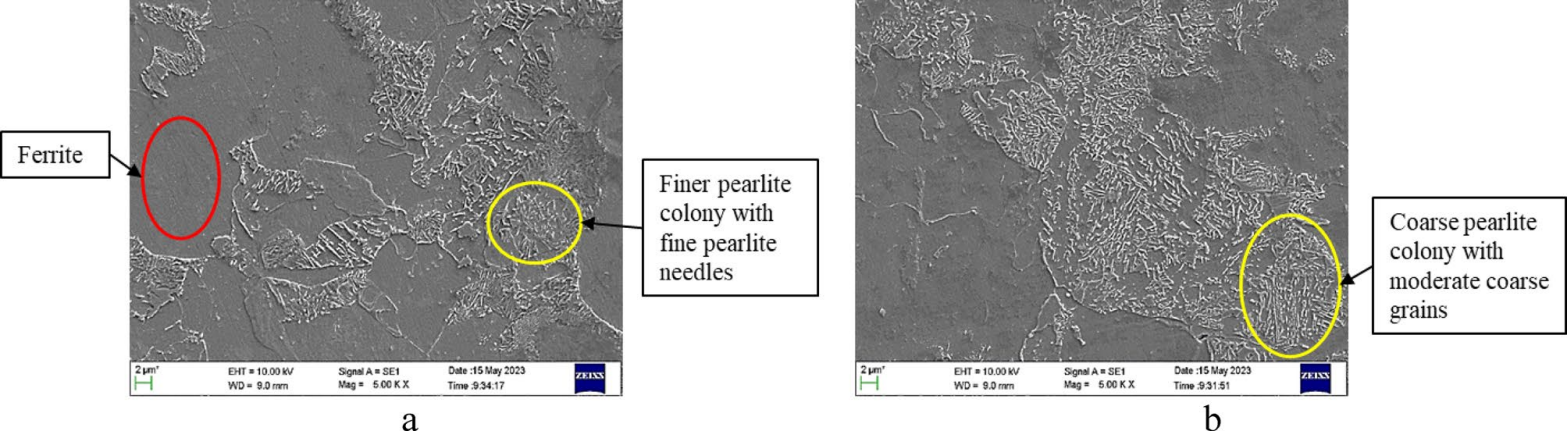
SEM image of AISI 4140 steel normalized at (**a**) 900 °C for 2 h and (**b**) 950 °C for 2 h.

**Fig. 6:**
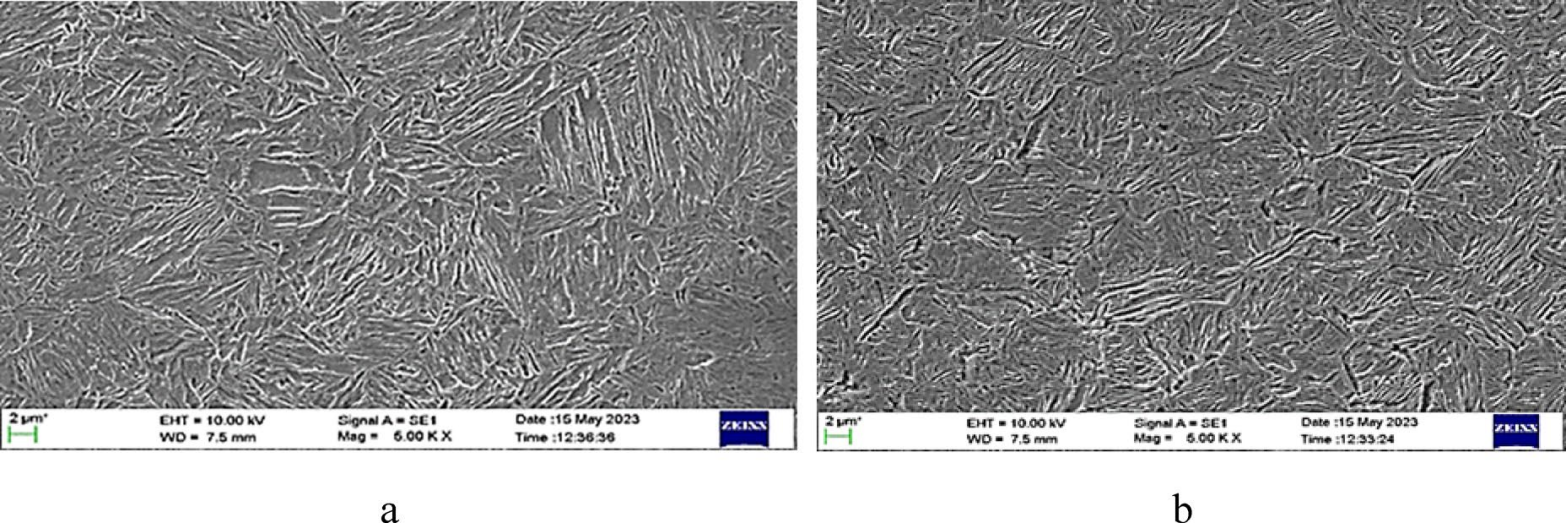
SEM images of AISI 4140 steel oil quenched at (**a**) 900 °C for 2 h, (**b**) 950 °C for 2 h.

The effect of heat treatment (annealing, normalizing and oil quenching) on the mechanical properties (hardness and impact energy) of the heat treated samples for AISI 4140 are as shown in Table 3. The results, based on the average of 5 trials, had a standard variation less than 3%. The respective microstructure results for each heat treatment are as shown in Figs. 4, 5 and 6.

**Table 3:** Hardness and impact test results for AISI 4140.

Temperature (°C)	Time (h)	Annealing		Normalizing		Oil quenching	
		Hardness (HV)	Impact energy (J)	Hardness (HV)	Impact energy (J)	Hardness (HV)	Impact energy (J)
900	1	210	30	290	40	485	22
900	1.5	205	36	287	45	480	23
900	2	203	43	284	48	478	24
925	1	200	38	280	45	450	26
925	1.5	195	42	275	48	420	28
925	2	190	47	270	50	400	30
950	1	190	44	250	50	360	36
950	1.5	185	49	230	55	320	38
950	2	183	54	219	60	286	42

Based on data from Table 3 and analysing the microstructure shown in Fig. 4, it may be seen that hardness of AISI 4140 steel decreases, and impact energy increases with increase in annealing temperature and time. At 900 °C (1–2 h), hardness drops from 210 HV to 203 HV; impact energy rises from 30 J to 43 J due to reduced dislocations and wider pearlite spacing. At 925 °C, hardness falls to 190 HV and impact energy reaches 47 J, with finer pearlite in ferrite matrix. At 950 °C, steel softens rapidly (183 HV, 54 J), showing coarse pearlite and large ferrite grains. Increased interlamellar spacing drives softening and toughness.

Normalizing for AISI 4140, as observed in Table 3; Fig. 5, at increasing temperatures and times, lowers hardness and boosts impact energy. At 900 °C (1–2 h), hardness drops from 290 HV to 284 HV; impact energy rises from 40 J to 48 J due to fine pearlite–ferrite structure. At 925 °C, hardness declines to 270 HV and impact energy reaches 50 J as microstructure becomes more uniform. At 950 °C, grain growth and coarse pearlite reduce hardness to 219 HV while impact energy peaks at 60 J. Higher temperature and time soften the steel while increasing toughness.

When AISI 4140 was subject to oil quenching heat treatment, as seen in Table 3; Fig. 6, it was observed that at 900 °C (1–2 h) martensitic structure was formed, giving high hardness (485 HV to 478 HV) and low toughness (22 J to 24 J). Longer soaking increases austenite grain size, producing coarser martensite and slightly reducing hardness while improving impact energy. Similar trends occur at 925 °C and 950 °C. Overall, higher temperature and time reduce hardness and increase toughness due to martensite coarsening.

### ANOVA

To assess the influence of heat treatment parameters, such as temperature and time, on the mechanical properties of AISI 4140 steel, a two-way ANOVA was conducted at a 95% confidence level, using MINITAB 17 statistical analysis software. Analysis of Variance (ANOVA) is a technique that evaluates statistical significance of factors and their interactions on measured responses by partitioning total variability into contributions from each source. This helps in identifing which parameters significantly influence the outcomes. The mechanical properties evaluated were Vickers microhardness and Charpy impact energy. The percentage contributions of each factor were also calculated to understand their relative influence on the properties.

The results of ANOVA of hardness and impact energy for AISI 4140 are as shown in table’s 4 and 5 respectively.

**Table 4:** ANOVA results of impact energy for AISI 4140.

Heat treatment	Factors	Adj. SS	Adj. Ms	F-Value	*P*-Value	% Contribution	*R*-sq %
Annealing	Temperature	602.000	301.000	301.00	<0.0001	85.51	99.43
	Time	98.000	49.000	49.00	0.002	13.92	
Normalizing	Temperature	4824.0	2412.00	50.96	0.001	89.56	96.48
	Time	372.7	186.33	3.94	0.113	6.92	
Oil quenching	Temperature	38875	19437.4	67.23	0.001	90.59	97.31
	Time	2880	1440.1	4.98	0.082	6.71	

The ANOVA results, as tabulated in table 4 and 5, show that for both hardness and impact energy heat treatment temperature is the greater influential factor. Temperature has nearly 85.5 % influence on the hardness of annealed specimens, 89.5 % on normalized and 90.5 % on oil quenched specimens. For impact energy, temperature has nearly 57.87 % influence on annealed specimens, 65.2 % on normalized and 93 % on oil quenched specimens. Even though holding time also has some influence on hardness and impact energy, its influence is less compared to heat treatment temperature.

**Fig. 7:**
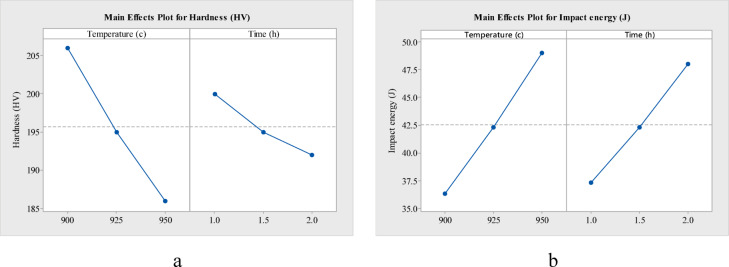
Main effect plot of (**a**) hardness and (**b**) impact energy, for annealing of AISI 4140.

**Fig. 8:**
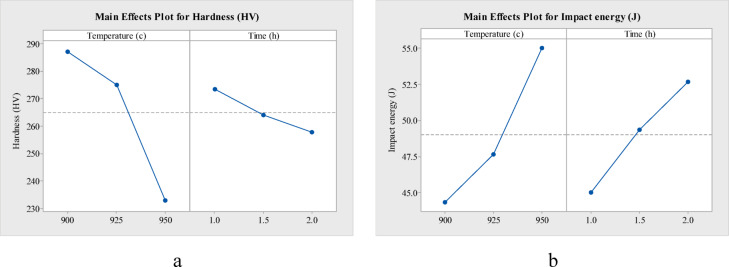
Main effect plot of (**a**) hardness and (**b**) impact energy, for normalizing of AISI 4140.

**Fig. 9:**
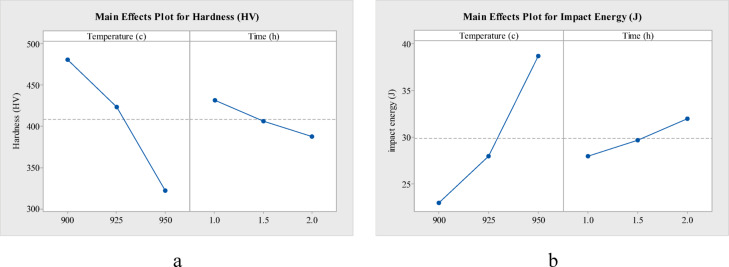
Main effect plot of (**a**) hardness and (**b**) impact energy, for oil quenching of AISI 4140.

The main effect plots of hardness and impact energy w.r.t temperature and time, for annealing (Fig. 7), normalizing (Fig. 8) and oil quenching (Fig. 9) of AISI 4140, depict a downward slope for hardness and an upward slope for impact energy. That is, as heat treatment temperature (900 to 950 °C) increases, there is nearly a 20 HV decrease in hardness for annealing, a 55 HV decrease in hardness for normalizing and around 160 HV drop in hardness for oil quenching. Whereas with an increase in temperature, impact energy increases by 10 to 15 J in each case. Holding time also has a similar effect on hardness and impact energy for each heat treatment.

**Table 5:** ANOVA results of impact energy for AISI 4140.

Heat treatment	Factors	Adj. SS	Adj. Ms	F-Value	*P*-Value	% Contribution	*R*-sq %
Annealing	Temperature	240.889	120.444	108.40	< 0.0001	57.87	98.93
	Time	170.889	85.444	76.90	0.001	41.06	
Normalizing	Temperature	178.667	89.333	53.60	0.001	65.23	97.57
	Time	88.667	44.333	26.60	0.005	32.36	
Oil quenching	Temperature	384.222	192.111	172.90	0.000	93.05	98.92
	Time	24.222	12.111	10.90	0.024	5.87	

### Regression analysis

To further understand and evaluate the effect of heat treatment temperature and holding time on hardness and impact energy, for each of the heat treatments, a multiple linear regression analysis was conducted.

The regression equations, with respective R-sq, R-sq (adj) and R-sq (pred) values, of hardness and impact energy for annealing are as shown below;

1 $${\text{Hardness }}\left( {{\text{HV}}} \right)\,=\,{\text{577}}.{\text{7}}\, - \,0.{\text{4}}000{\text{ Temperature }}\left( {\text{c}} \right) - \,{\text{8}}.000{\text{ Time }}\left( {\text{h}} \right)$$

R-sq = 98.86%; R-sq (adj) = 98.48%; R-sq (pred) = 97.15%

2 $${\text{Impact energy }}\left( {\text{J}} \right){\text{ }}={\text{ }} - {\text{2}}0{\text{7}}.{\text{8}}\,+\,0.{\text{2533 Temperature }}\left( {\text{c}} \right)\,+\,{\text{1}}0.{\text{667 Time }}\left( {\text{h}} \right)$$

R-sq = 98.83%; R-sq (adj) = 98.43%; R-sq (pred) = 96.80%.

Regression Eqs. (1) and (2) can be used to evaluate the hardness and impact energy of the annealed AISI 4140 steel for the heat treatment parameters which are within the range of values considered for current study.

Similarly, for normalizing;

3 $${\text{Hardness }}\left( {{\text{HV}}} \right)\,=\,{\text{1288}}\, - \,{\text{1}}.0{\text{8}}0{\text{ Temperature }}\left( {\text{c}} \right) - \,{\text{15}}.{\text{67 Time }}\left( {\text{h}} \right)$$

R-sq = 88.05%; R-sq (adj) = 84.06%; R-sq (pred) = 72.43%

4 $${\text{Impact energy }}\left( {\text{J}} \right){\text{ }}={\text{ }} - {\text{159}}.{\text{8}}\,+\,0.{\text{2133 Temperature }}\left( {\text{c}} \right)\,+\,{\text{7}}.{\text{67 Time }}\left( {\text{h}} \right)$$

R-sq = 94.46%; R-sq (adj) = 92.62%; R-sq (pred) = 87.75%.

Regression Eqs. (3) and (4) can be used to evaluate the hardness and impact energy of the normalized AISI 4140 steel for the heat treatment parameters which are within the range of values considered for current study.

And for oil quenching;

5 $${\text{Hardness }}\left( {{\text{HV}}} \right)\,=\,{\text{3416}} - {\text{3}}.{\text{18}}0{\text{ Temperature }}\left( {\text{c}} \right){\text{ }} - \,{\text{43}}.{\text{7 Time }}\left( {\text{h}} \right)$$

R-sq = 95.04%; R-sq (adj) = 93.38%; R-sq (pred) = 86.66%

6 $${\text{Impact Energy }}\left( {\text{J}} \right){\text{ }}={\text{ }} - {\text{265}}.{\text{9}}\,+\,0.{\text{3133 Temperature }}\left( {\text{c}} \right)\,+\,{\text{4}}.00{\text{ Time }}\left( {\text{h}} \right)$$

R-sq = 94.98%; R-sq (adj) = 93.31%; R-sq (pred) = 88.04%.

Regression Eqs. (5) and (6) can be used to evaluate the hardness and impact energy of the oil quenched AISI 4140 steel for the heat treatment parameters which are within the range of values considered for current study.

### Multiple response optimization

Multiple response optimization was done to get the optimal temperature and time for best combination of hardness and impact energy for each heat treatment. This method of optimization determines process settings that simultaneously satisfy desired targets for multiple responses. Based on optimization results obtained through response surface optimization analysis, with an aim to get superior combination of hardness and impact energy, following combination of parameters, as shown in Table 6, were found to be optimal.

**Table 6:** Response optimization results.

Heat treatment	Optimal temperature (°C)	Optimal time (h)	Hardness (HV)	Impact energy (J)	Composite desirability index (D)
Annealing	900	2	202	42.5	0.6114
Normalizing	919.7	2	274	50	0.6284
Oil quenching	944.95	1	382.25	33.37	0.5243

For annealing at the optimal temperature and time of 900 °C and 2 h, the best combination of hardness (202 HV) and impact energy (42.5 J) was obtained. For normalizing, the optimized values were found to be 919.7 °C and 2 h, for a hardness and impact energy values of 274 HV and 50 J respectively. Similarly for oil quenching, at 944.95 °C and 1 h temperature and time, the best combination of hardness (382.25 HV) and impact energy (33.37 J) was obtained. The composite desirability indexes, as shown in Table 6, indicate a balance between the two competing objectives. While the values are not close to 1 (ideal desirability), they reflect a feasible compromise between maximizing both mechanical properties.

## Conclusion

The effects of annealing, normalizing, and oil quenching on the hardness and impact energy of AISI 4140 steel over an austenitization heat treatment temperature range of 900–950 °C and holding times of 1–2 h, was studied. It was observed that for all the three heat treatments, hardness decreased, and impact energy increased with higher heat treatment temperature and longer holding time, with the rate of hardness loss per °C/h highest for oil quenching (3.98 HV), followed by normalizing (1.42 HV) and annealing (0.54 HV). Based on the ANOVA results from Tables 4 and 5 we can infer that heat treatment temperature has the greater influence, ranging from 57.87 to 93%, while holding time had a lesser influence (5.87 to 41%) on hardness and impact energy of the steel, across all three heat treatment conditions.

Based on the results obtained, the following conclusions were made:

- Oil quenching produced the highest hardness, averaging 120% higher than annealing and 58.63% higher than normalizing, but with the lowest impact energy. But as the heat treatment temperature increased from 900 °C to 950 °C and holding time from 1 to 2 h, hardness decreased at a rate of 3.98 HV/°C/h, and impact energy increased at a rate of 0.4 J/°C/h.
- Annealing had the lowest hardness but the highest relative gain in toughness, with impact energy increasing by 0.48 J/°C/h and hardness decreasing at a rate of 0.54 HV/°C/h.
- While normalizing offered balanced properties, with a 39% increase in hardness over annealing and a 17.5% increase in impact energy, hardness decreased at a rate of 1.42 HV/°C/h and impact energy increased by 0.4 J/°C/h with increase in heat treatment temperature and holding time.
- The ANOVA results show that heat treatment temperature had a greater significant effect on the hardness (90.59%) and impact energy (93.05%) of the steel when it was subjected to oil quenching, while for annealing it had the lowest, 85.51% for hardness and 57.87% for impact energy. While holding time had the most effect on hardness (13.92%) and impact energy (41.06%) for annealing.
- The optimization results showed that a temperature of 900 °C and holding time of 2 h gave the best combination of hardness and impact energy for annealed specimens, 919.7 °C and 2 h for normalized specimens, and 944.95 °C and 1 h for oil quenched specimens.

These findings show a trade-off between strength and toughness in heat treatment design and provide quantitative guidance for selecting parameters to meet specific performance requirements.

## Data Availability

Corresponding author agrees to provide the data upon reasonable request.
